# Predictors of Non-Cardiovascular Readmissions in Multimorbid Adults with Heart Failure in Australian Hospitals: A Retrospective Cohort Study

**DOI:** 10.3390/jcm15135275

**Published:** 2026-07-06

**Authors:** Joshua M. Inglis, Gillian E. Caughey, Tracy Air, John Maddison, Danny Liew, Sepehr Shakib

**Affiliations:** 1 School of Pharmacy and Biomedical Sciences, College of Health, Adelaide University, Adelaide, SA 5005, Australia; 2 Department of Clinical Pharmacology, Flinders Medical Centre and Flinders University, Bedford Park, SA 5042, Australia; 3 Department of General Medicine, Royal Adelaide Hospital, Adelaide, SA 5000, Australia; 4 Registry of Senior Australians Research Centre, South Australian Health and Medical Research Institute, Adelaide, SA 5000, Australia; 5 Registry of Senior Australians Research Centre, Caring Futures Institute, College of Nursing and Health Sciences, Flinders University, Bedford Park, SA 5042, Australia; 6 Northern Adelaide Local Health Network, Elizabeth Vale, SA 5112, Australia; 7 Faculty of Health, Medicine and Behavioural Sciences, The University of Queensland, Brisbane, QLD 4072, Australia

**Keywords:** multimorbidity, heart failure, medications, polypharmacy, readmissions

## Abstract

**Background:** Heart failure is common in hospitalised adults, who often have high rates of polypharmacy. Two-thirds of readmissions are for non-cardiovascular reasons, where medications and comorbidities are likely to be contributors. The aim of this study was to quantify the proportion of non-cardiovascular readmissions and examine the factors associated with non-cardiovascular readmissions among multimorbid adults with heart failure. **Methods:** We conducted a retrospective cohort study of multimorbid adults aged ≥ 45 years with heart failure who were discharged from four metropolitan hospitals between August 2016 and June 2023. Non-cardiovascular polypharmacy was defined as the use of five or more non-cardiovascular medications as extracted from the electronic medical record at discharge from the hospitals. Non-cardiovascular readmissions were defined as those with a non-cardiovascular condition as the principal diagnosis. The proportion of patients with one or more non-cardiovascular readmissions, cardiovascular readmissions, or deaths by 12 months was calculated. Logistic regression was used to determine the factors associated with the occurrence of a non-cardiovascular readmission over 12 months. **Results:** In total, 4912 patients were included in the study, with 56% (n = 2734) having non-cardiovascular polypharmacy. The proportion of patients with one or more non-cardiovascular readmissions was 32% at 12 months post-discharge. Nineteen percent of patients had one or more cardiovascular readmissions, while a further 28% had died by 12 months. Non-cardiovascular polypharmacy was associated with a 48% increase in the odds (OR 1.48 95% CI 1.28–1.72) of having a non-cardiovascular readmission within 12 months. **Conclusions:** Non-cardiovascular readmissions occurred more frequently than cardiovascular readmissions in adults with heart failure. Non-cardiovascular polypharmacy was associated with a 48% increase in the odds of having a non-cardiovascular readmission, potentially through medication-related harm or as a surrogate of comorbidity burden. Future studies should explore holistic approaches to the management of multimorbid adults with heart failure, including optimisation of comorbidities and non-cardiovascular medications.

## 1. Introduction

Heart failure is a common medical condition affecting 64 million people worldwide [1]. In Australia, there are 144,000 people living with heart failure who have 173,300 heart failure-related hospitalisations each year, representing 1.5% of all hospital admissions [2]. Heart failure is associated with premature mortality, increased hospital utilisation, poor functional status and reduced quality of life [3]. Approximately one-third of hospitalised heart failure patients are readmitted within 30 days of discharge, incurring $600 million of direct hospital costs per year in Australia [4]. Two-thirds of these readmissions are for non-cardiovascular reasons, and approximately half of all readmissions are potentially preventable [4]. While many readmission prevention interventions have been developed, these largely focus on heart failure, with few targeting readmissions for non-cardiovascular reasons [5]. An understanding of the factors associated with non-cardiovascular readmissions in those with heart failure is needed to develop targeted interventions to reduce preventable non-cardiovascular readmissions.

While guideline-directed medicines have been shown to prevent hospitalisations for heart failure, the factors associated with the more common non-cardiovascular hospitalisations are less clear. Medication-related harms such as injurious falls, bleeding, delirium, dehydration and renal impairment are common causes of preventable hospitalisation in multimorbid adults, including those with heart failure [6,7]. Furthermore, it has been shown that potential drug-disease interactions are common in older adults with heart failure who have multiple chronic conditions [8]. The use of potentially inappropriate non-cardiovascular medicines may be associated with non-cardiovascular readmissions due to the potential for medication-related harms (e.g., adverse drug reactions, drug interactions) and as an indicator of the underlying comorbidity burden. Understanding the association of medication-related factors with hospital readmissions may identify potential targets to optimise medication use and prevent medication-related harm [9]. This study aimed to quantify the proportion of non-cardiovascular readmissions and to examine the factors associated with non-cardiovascular readmissions among multimorbid adults with heart failure.

## 2. Method

### 2.1. Study Type and Setting

A retrospective cohort study reported in accordance with the RECORD statement [10] was conducted using hospital admission data from the Royal Adelaide Hospital, the Queen Elizabeth Hospital, the Lyell McEwin Hospital and Modbury Hospital in Adelaide, South Australia, between August 2016 and June 2022, with follow-up to June 2023. This included a total of 198,000 annual inpatient admissions (95,000 per annum at the Royal Adelaide Hospital, 40,000 at the Queen Elizabeth Hospital, 45,000 at Lyell McEwin Hospital and 18,000 at Modbury Hospital) [11]. These hospitals use a modification of the Altera Digital Health Sunrise electronic medical record (Altera Digital Health, Niagara Falls, NY, USA). There was no data linkage. All first hospitalisations during the study period were included, so no further sampling strategy was applied.

### 2.2. Study Cohort

The inclusion criteria were first hospital admissions for adults aged ≥ 45 years with heart failure and multimorbidity during the study period. Heart failure was identified using select ICD-10 codes (I50.0, I50.1 or I50.9) during the index admission as either a principal or supplementary diagnosis. This definition was chosen to capture both patients admitted for heart failure and those admitted for other reasons (e.g., respiratory tract infections) that would not have resulted in an admission if the patient did not have heart failure. Furthermore, the presence of an ICD-10 code for heart failure in the Australian setting indicates that the condition was actively managed during the admission. These ICD-10 codes have been shown to have a 96.5% agreement with the presence of chronic heart failure as documented in the medical records in the Australian setting [12]. Multimorbidity was defined as two or more comorbidity groups, identified using the pharmaceutical-based comorbidity index (RxRisk V) [13]. This comorbidity index was chosen because it has the highest sensitivity (60.4%) and a positive predictor value (62.5%) for identifying multimorbidity compared to the gold standard of self-reported conditions when using routinely collected data in the Australian setting [14]. We chose this measure in favour of other methods that had a slightly higher sensitivity but a lower positive predictor value to ensure that our cohort truly represented patients with multimorbidity.

All unplanned acute hospital admissions were in specific units that manage patients with general comorbidities, such as General Medicine, General Surgery and Geriatrics, and patients were required to have a length of inpatient stay ≥48 h to allow sufficient time for preadmission medications to be charted into the EMR. Admissions where the patient did not survive or were transferred to another hospital were excluded.

### 2.3. Study Variables

The study variables were age, gender, hospital admission ICD-10 codes and regular medication orders at the time of discharge, which were extracted from the EMR. ICD-10 codes from the index hospital admission were used to calculate the Charlson comorbidity index (excluding heart failure, given that all individuals in the cohort had this condition) [15]. Medicine orders were mapped to the World Health Organization Anatomical Therapeutic Chemical (ATC) classification system [16]. Regular medications were defined as medication orders in the EMR at the time of discharge, excluding short-term and as-needed medications. Not included were prophylactic orders for enoxaparin, all intravenous, subcutaneous and intramuscular medicines other than those for long-term use (e.g., insulins, antiresorptives, glucagon-like peptide-1 receptor agonists and erythrocyte-stimulating agents), as well as anti-infectives (ATC Code J). ATC codes were used to classify cardiovascular medicines (C01-C10), antithrombotics and antiplatelets (B01), SGLT2i inhibitors (A10BJ) and GLP-1 receptor agonists (A10BK), and non-cardiovascular medicines (ATC Classes D-V, Class A excluding A10BJ and A10BK, and Classes B2-B6). Non-cardiovascular polypharmacy was defined as five or more unique non-cardiovascular medications classified using ATC codes in accordance with the accepted definition for polypharmacy [17]. The rationale for this definition is that polypharmacy (defined as five or more medications) has been associated with adverse health outcomes, including hospitalisation in a range of populations [17,18,19]. Cardiovascular medications in heart failure have generally been associated with reductions in hospitalisation, unlike non-cardiovascular medications [20]. Furthermore, non-cardiovascular medications have the potential to cause non-cardiovascular readmissions through medication-related harm such as falls, delirium, dehydration and acute kidney injury [7]. Therefore, it was hypothesised that non-cardiovascular polypharmacy would be associated with all-cause and non-cardiovascular readmissions.

Hospital visits and coding data using the ICD-10-Australian Modification were extracted for the index and subsequent admissions. This included the dates of admission and discharge. The Charlson comorbidity index was calculated using ICD-10 codes from the index admission, excluding heart failure [15]. The hospital frailty risk score, which uses ICD-10 codes for markers of frailty (e.g., cognitive impairment, functional dependence, falls and fractures), was used to identify those with frailty [21]. Individuals with an intermediate or high risk of frailty using this score were considered to be frail, consistent with the previous studies.

### 2.4. Study Outcomes

Acute hospital readmissions were identified from study entry (index admission) up to 1 year following discharge at 3 monthly intervals (Supplementary S1). Readmission due to non-cardiovascular reasons was also identified by those with a non-cardiovascular principal diagnosis (ICD-10 code in the first position). Date of death was obtained from the South Australian Births, Deaths and Marriages registry.

### 2.5. Statistical Analysis

Descriptive statistics were calculated for baseline characteristics of the study cohort, including age, gender, Charlson comorbidity index excluding heart failure, RxRisk comorbidity score excluding heart failure, frailty status, number of regular medicines, number of cardiovascular medicines, number of non-cardiovascular medicines, and the presence of non-cardiovascular polypharmacy. The proportion of patients with one or more non-cardiovascular or cardiovascular readmission and the number of each readmission type was also reported alongside mortality at 12 months.

The cumulative incidence of first all-cause readmission or death was calculated at 3 monthly intervals up to 1 year. This was estimated using the Aalen–Johansen method to account for competing risks, implemented using the *cmprsk* package in RStudio [22]. The number of events within each category at 1 year has been reported as percentages with 95% confidence intervals. These were stratified by the presence of non-cardiovascular polypharmacy.

Fine and Grey’s regression was used to determine the association of non-cardiovascular polypharmacy with all-cause readmissions up to 12 months, accounting for the competing risk of death. Factors examined in the model included age, gender, score of cardiovascular conditions in the Charlson comorbidity index (excluding heart failure), and the presence of non-cardiovascular polypharmacy. These were presented as subdistribution hazard ratios (sHRs) with 95% confidence intervals.

Logistic regression was used to determine the factors associated with the occurrence of non-cardiovascular readmissions within 12 months for those who survived to 12 months. Logistic regression was used (rather than time-to-event analyses), given the aim was to assess the factors associated with non-cardiovascular readmissions rather than the time to a non-cardiovascular readmission. Additionally, cardiovascular readmissions are not a competing risk (unlike death), as they do not preclude future non-cardiovascular readmissions. Factors examined in the logistic regression included age, gender, score of cardiovascular comorbidities in the Charlson comorbidity index, excluding heart failure, and the presence of non-cardiovascular polypharmacy.

In both of these analyses, chronic heart failure was excluded from the Charlson comorbidity index, given that all individuals in the cohort had this condition. Only cardiovascular comorbidities (excluding heart failure) from the Charlson comorbidity index were included to minimise collinearity between the comorbidity burden and non-cardiovascular polypharmacy, as both were considered measures of the non-cardiovascular comorbidity burden (Supplementary S2) [15]. Similarly, the hospital frailty risk score was not included as a covariate given the overlap with the Charlson comorbidity index since both were calculated from ICD-10 codes.

Missing data were minimal (<5%) due to the structured nature of the electronic medical record. Variables were assessed for collinearity using the variance inflation factor (VIF). All VIFs were less than 6, indicating that there was no significant collinearity between the covariates. The proportional hazards assumption was assessed through visual inspection of diagnostic plots with no violations identified. No other sensitivity analyses were performed, as results were expected to be robust given the low proportion of missing data and inclusion of all eligible hospitalisations with prescribing data.

Analysis was performed using RStudio, version 4.1.2 (R Foundation for Statistical Computing, Vienna, Austria).

### 2.6. Ethics

This study was approved by the SA Health Department of Health and Wellbeing Human Research Ethics Committee (2023/HRE00019).

## 3. Results

### 3.1. Overall Study Cohort Characteristics

**Figure 1:**
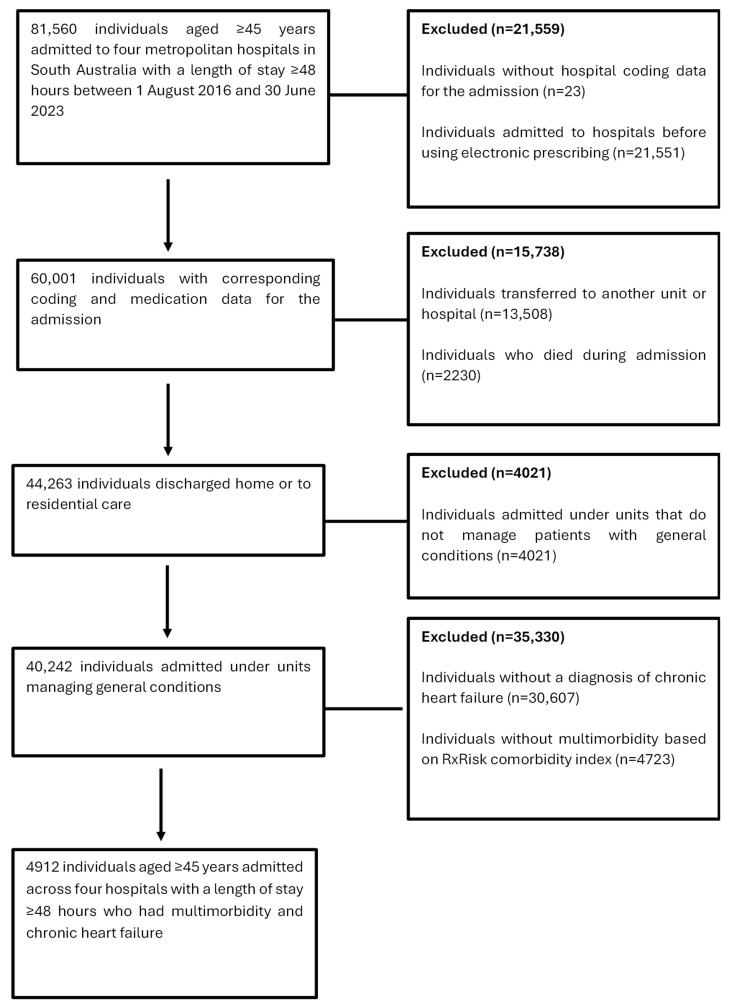
Flow chart for study cohort selection.

This study included 4912 individuals with heart failure and multimorbidity discharged from hospital to home or residential care during the study period (Table 1 and Figure 1). The median age was 82 years (IQR 73–89), and 49% were female (n = 2394). Patients had a median of 6 comorbidity groups, excluding chronic heart failure (IQR 5–8), based on the pharmaceutical comorbidity index. The median Charlson comorbidity score (excluding heart failure) was 3 (IQR 2–4). Fifty-eight percent (n = 2863) of patients were frail based on the hospital frailty risk score.

Ninety-five percent (n = 4662) of patients had polypharmacy, and fifty-two (n = 2578) had hyperpolypharmacy. The median number of medications was 10 (IQR 7–12). Fifty-six percent (n = 2734) of patients had non-cardiovascular polypharmacy. Patients were taking a similar number of cardiovascular (median 5, IQR 3–6) and non-cardiovascular medicines (median 5, IQR 3–8).

Twenty-eight percent (n = 1367) of the cohort died within 12 months. Thirty-two percent (n = 1578) had one or more non-cardiovascular readmissions, and 19% (n = 935) had one or more cardiovascular readmissions within 12 months.

Of all patients in the study cohort, 7.7% (n = 380) had one non-cardiovascular readmission, 8.3% (n = 407) had 2–3 non-cardiovascular readmissions, and 16% (n = 791) had ≥4 non-cardiovascular readmissions within 12 months. Of all patients in the study cohort, 7.4% (n = 363) had one cardiovascular readmission, 5.7% (n = 281) had 2–3 cardiovascular readmissions, and 5.9% (n = 291) had ≥4 cardiovascular readmissions within 12 months.

### 3.2. Prevalence of Comorbidities of Chronic Heart Failure

**Figure 2:**
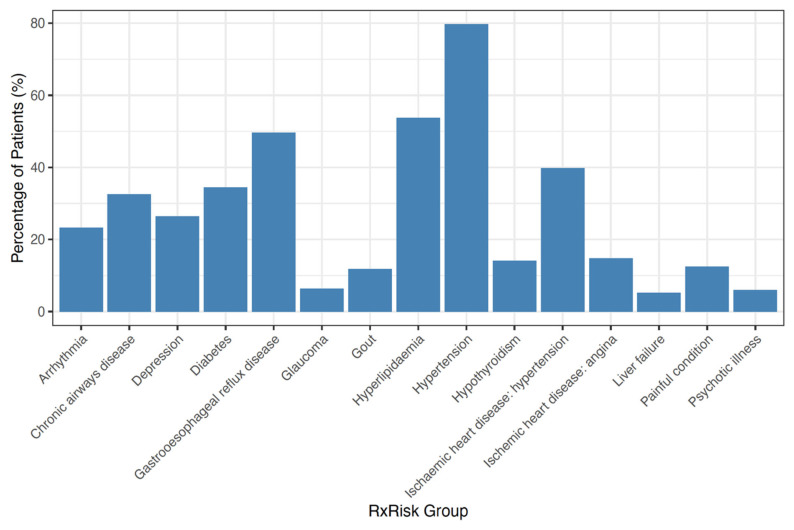
Prevalence of comorbid conditions in hospitalised multimorbid adults ≥ 45 years with heart failure. (Only those comorbid conditions with a prevalence of ≥5% are presented.).

The most common pharmaceutical comorbidity groups based on the RxRisk pharmaceutical comorbidity index were hypertension (79.7%, n = 3914), hyperlipidaemia (53.8%, n = 2642), gastro-oesophageal reflux disease (49.6%, n = 2436), ischaemic heart disease with hypertension (39.8%, n = 1957), diabetes (34.5%, n = 1694), chronic airways disease (32.5%, n = 1596), depression (26.4%, n = 1299), arrhythmia (23.3% n = 1145), ischemic heart disease with angina (14.7%, n = 724), hypothyroidism (13.3%, n = 653), painful conditions (12.5%, n = 613), gout (11.8%, n = 581), glaucoma (6.3%, n = 311), psychotic illness (5.9%, n = 292), and liver failure (5.2%, n = 256) (Figure 2).

### 3.3. Cumulative Incidence of All-Cause Readmissions and Death

**Figure 3:**
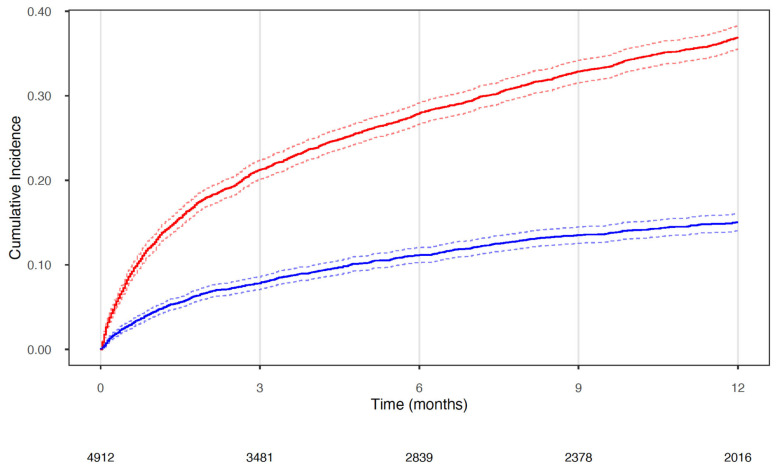
Cumulative incidence of time to first all-cause readmission or death in multimorbid adults with chronic heart failure. The number at risk is indicated at the bottom.

Figure 3 presents the cumulative incidence of time to first all-cause readmission or death. The cumulative incidence of all-cause readmissions was 21.2% (95% CI 20.1–22.4%) at 3 months, 27.9% (95% CI 26.6–29.1%) at 6 months, 32.8% (95% CI 31.5–34.2%) at 9 months and 36.9% (95% CI 35.5–38.3%) at 12 months. The risk of death was 7.8% (95% CI 7.1–8.6%) at 3 months, 11.2% (95% CI 10.3–12.0%) at 6 months, 13.5% (95% CI 12.6–14.5%) at 9 months and 15.0% (95% CI 14.0–16.1%) at 12 months.

### 3.4. Cumulative Incidence of All-Cause Readmissions and Death Stratified by Non-Cardiovascular Polypharmacy

**Figure 4:**
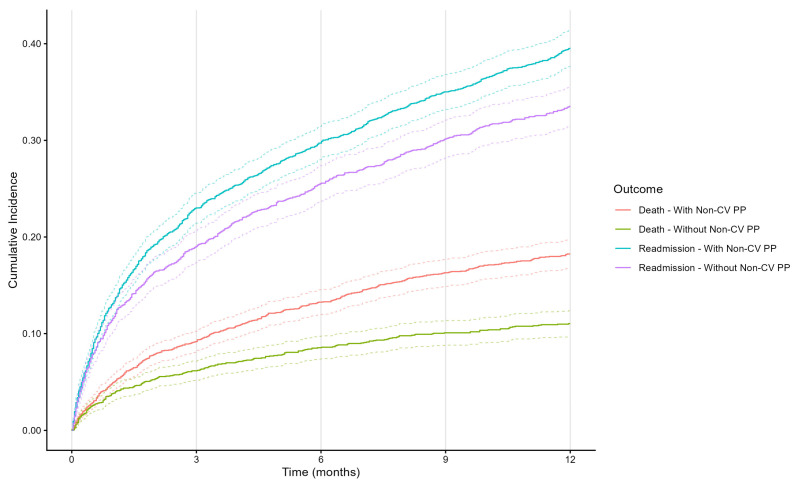
Cumulative incidence of time to first all-cause readmission or death in multimorbid adults with chronic heart failure stratified by non-cardiovascular polypharmacy. (Abbreviations: CV—cardiovascular, PP — polypharmacy).

Figure 4 presents the cumulative incidence of time to first all-cause readmission or death stratified by the presence or absence of non-cardiovascular polypharmacy. The cumulative incidence of all-cause readmissions at 12 months was 39.2% (95% CI 37.5–40.9%) in those with non-cardiovascular polypharmacy compared to 31.9% (95% CI 29.5–34.2%) without non-cardiovascular polypharmacy. The cumulative incidence of death at 12 months was 17.4% (95% CI 16.1–18.7%) with non-cardiovascular polypharmacy compared to 10.0% (95% CI 8.5–11.5) without non-cardiovascular polypharmacy.

### 3.5. Factors Associated with Time to First All-Cause Readmission at 12 Months

Fine and Grey’s regression demonstrated that non-cardiovascular polypharmacy was associated with an increased risk of all-cause readmission within 12 months (sHR 1.22 95% CI 1.11–1.34) (Table 2). Each additional point for cardiovascular conditions in the Charlson comorbidity index increased the risk of all-cause readmissions (sHR 1.32 95% CI 1.20–1.44). Both the age 75–84 years (sHR 1.37 95% CI 1.04–1.80) and the age 85 years and over bands (sHR 1.43 95% CI 1.09–1.87) were associated with an increased risk when compared to the age 45–54 years band. There was no association with gender or the remaining age bands.

### 3.6. Factors Associated with Non-Cardiovascular Readmissions at 12 Months

Logistic regression demonstrated that non-cardiovascular polypharmacy was associated with a 48% increase in the odds of having a non-cardiovascular readmission within 12 months in those who survived this period (OR 1.48 95% CI 1.28–1.72) (Table 3). Each additional point for cardiovascular conditions in the Charlson comorbidity index increased the odds of having a non-cardiovascular readmission within 12 months by 58% (OR 1.58 95% CI 1.36–1.82). There was no association with age bands or gender. An aggregate list of principal diagnoses for first non-cardiovascular readmissions is included in Supplementary Table S1.

## 4. Discussion

This study describes the burden of all-cause, non-cardiovascular and cardiovascular readmissions, and examines the factors associated with non-cardiovascular readmissions among multimorbid adults with heart failure. Readmissions for non-cardiovascular reasons were the most common outcome in this cohort of multimorbid adults with heart failure. Thirty-two percent of admitted adults with heart failure had one or more non-cardiovascular readmissions by 12 months, compared to 19% who had a cardiovascular readmission and 28% who died within 12 months. Non-cardiovascular polypharmacy was associated with a 48% increase in the odds of having a non-cardiovascular readmission by 12 months, and 56% of patients in the cohort were exposed to non-cardiovascular polypharmacy. This may reflect the contribution of either non-cardiovascular medications or comorbidities to readmissions for multimorbid adults with heart failure. These findings suggest that interventions should address a wide range of post-discharge risks beyond cardiovascular complications, including those related to medications and comorbidities. The high burden of non-cardiovascular medications and their association with non-cardiovascular readmissions in admitted multimorbid adults with heart failure highlights the need for medication and comorbidity optimisation to be incorporated into the routine care of these patients.

The rate of polypharmacy (95%) in hospitalised multimorbid adults with chronic heart failure is within the estimates from similar populations in the Australian setting (60–95%). One Australian study from 2007 found that 98.7% of older veterans with heart failure had polypharmacy [8]. Other Australian studies have shown that 76.9% of adults admitted with acute decompensated heart failure and 83.7% of admitted adults with systolic heart failure had polypharmacy [23,24]. This is similar to international estimates in hospitalised populations showing that 60–95% have polypharmacy [25,26,27] and 69–76% have hyperpolypharmacy [25,27]. The high rate of medication use in this group may confer an increased risk of medication-related harm or indicate comorbid conditions requiring optimisation that could otherwise lead to non-cardiovascular readmissions.

This study assesses factors associated with non-cardiovascular readmissions within the first 12 months after discharge. Many studies to date have focused on earlier time points for readmissions (e.g., within 30 days and 90 days), which may be more reflective of the quality of care provided on discharge [28,29]. In contrast, 12 months was chosen in this study as a better time point to assess the contribution of medications or non-cardiovascular comorbidities towards readmissions. Non-cardiovascular readmissions have been shown to be the most common readmission type in multimorbid adults with heart failure [4]. This may be partly due to the current emphasis on heart failure management in these patients. The reasons for non-cardiovascular readmissions in heart failure from previous studies have been related to both medications and comorbidities [30]. For instance, acute kidney injury, hypotension, fluid and electrolyte disorders, syncope, collapse, falls, and fractures were reasons for readmissions that were potentially related to medications. Additionally, a previous study has shown that non-cardiovascular readmissions from comorbidities are often due to chronic obstructive pulmonary disease and type 2 diabetes [30]. Notably, readmissions due to respiratory disorders were the most common principal diagnosis in this study.

Non-cardiovascular polypharmacy was associated with 48% higher odds of non-cardiovascular readmissions. This could be due to medication-related admissions or the underlying comorbidity burden. Non-cardiovascular medications are a recognised cause of medication-related admissions in multimorbid adults, including those with heart failure. In a recently published trigger tool developed from the OPERAM trial, non-cardiovascular medications led to admissions through falls, delirium, dehydration and acute kidney injury [6]. Therefore, this association could be the result of medication-related harm from non-cardiovascular medications causing readmissions. An alternative explanation is that non-cardiovascular polypharmacy is a surrogate for comorbidities that are associated with readmissions, such as chronic obstructive pulmonary disease and type 2 diabetes. It remains unclear whether the optimisation of medications or comorbidities at transitions of care would alter this risk, although pharmacist-led interventions have shown promise. An Australian clustered randomised controlled trial in recently hospitalised adults with chronic heart failure or chronic obstructive pulmonary disease showed that follow-up by a pharmacist and general practitioner within a week of discharge reduced readmissions and emergency department visits compared to usual care [31]. This may have occurred through improved management of heart failure, comorbidities or medication optimisation.

Future research is needed to better understand the factors contributing to non-cardiovascular readmissions in heart failure and to develop holistic interventions to prevent these readmissions. A large range of interventions has been proposed to address the high rate of readmissions in heart failure, which usually focus on management of the primary disease [32,33,34]. This study has shown that in a typical, elderly, multimorbid cohort of patients with heart failure, non-cardiovascular readmissions were the most common reason for readmissions. Given that the largest burden of readmissions is for non-cardiovascular reasons and non-cardiovascular polypharmacy is associated with this outcome, the contribution of non-cardiovascular medications and comorbidities should be the focus of future research [2,4]. This is needed to better understand the contributing factors for these readmissions with a view to developing better holistic interventions that address non-cardiovascular medications and comorbidities. These holistic interventions may have a much greater impact on patients and the health care system rather than focusing on further improvement in the management of heart failure or related cardiovascular conditions.

The strength of this study is that it is a large population of hospital inpatients reflecting real-world practices and outcomes. However, there are multiple limitations that should be acknowledged. Firstly, the use of principal diagnoses from hospital coding data may lack sensitivity or specificity for the detection of non-cardiovascular reasons for readmission. Secondly, medication orders at the time of hospital discharge may not equate to long-term medication use. Thirdly, the inability to include as-needed medications in the count of medications may have underestimated the rates of polypharmacy, although this is standard practice in studies using routinely collected data. Fourthly, there may be unmeasured confounding in the logistic regressions to determine the factors associated with non-cardiovascular readmissions. Heart failure type (i.e., reduced or preserved ejection fraction), New York Heart Association class, non-cardiovascular comorbidities, socioeconomic status, frailty status, ethnicity and discharge destination were not included as variables in the regressions, and this may have introduced confounding. The main implication is that the associations between non-cardiovascular polypharmacy and readmissions may reflect unmeasured non-cardiovascular comorbidity burden. Furthermore, the presence of frailty, lower socioeconomic status and discharge to residential care are associated with the use of a greater number of medications that may have attenuated the associations seen if these variables had been available and included as covariates. Frailty was not included as this was assessed using ICD-10 codes that were already being included through the use of the Charlson comorbidity index. Fifthly, the use of logistic regression with binary outcomes over a 12-month period does not account for differences in event timing or the competing risks over the follow-up period, unlike time-to-event analysis methods. However, this method was chosen given that these are not competing risks (cardiovascular readmissions do not preclude future non-cardiovascular readmissions) and the aim to assess the factors associated with these readmissions (rather than time to readmission). Sixthly, the generalisability of these findings to specialist hospital units and other settings, including regional or private hospitals, community-dwelling adults and international health systems, is unclear.

In an older multimorbid cohort with heart failure, 32% percent of patients had one or more non-cardiovascular readmissions by 12 months compared to 19% who had a cardiovascular readmission and 28% who died within 12 months. Non-cardiovascular polypharmacy was associated with a 48% increase in the odds of having a non-cardiovascular readmission within 12 months. These medications may be associated with non-cardiovascular readmissions through causing medication-related harm or may be a surrogate for the underlying comorbidity burden. This study highlights the potential benefit of medication and comorbidities optimisation to prevent non-cardiovascular readmissions in multimorbid adults with heart failure. Future studies are needed to determine whether a holistic approach to the management of multimorbid adults with heart failure could be implemented in the Australian setting to prevent non-cardiovascular readmissions. The findings of these studies would assist health services in optimising the models of care for multimorbid adults living with heart failure.

## Figures and Tables

**Table 1:** Baseline characteristics of hospitalised adults with multimorbidity and chronic heart failure.

Characteristic	All Patients N = 4912 ^1^
Age, median (IQR)	82 (73, 89)
Age band, n (%)	
45–54	200 (4.1%)
55–64	418 (8.5%)
65–74	771 (16%)
75–84	1482 (30%)
85+	2041 (42%)
Gender, n (%)	
Female	2394 (49%)
Male	2518 (51%)
RxRisk score minus CHF, median (IQR)	6 (5, 8)
Charlson conditions count minus CHF, median (IQR)	3 (2, 4)
Number of cardiovascular conditions in Charlson comorbidity index minus CHF, n (%)	
0	3807 (78%)
1	1021 (21%)
2–3	84 (1.7%)
Number of medications, median (IQR)	10 (7, 12)
Polypharmacy (≥5 medications), n (%)	4662 (95%)
Hyperpolypharmacy (≥10 medications), n (%)	2578 (52%)
Non-cardiovascular polypharmacy (≥5), n (%)	
No	2178 (44%)
Yes	2734 (56%)
Non-cardiovascular medications count, median (IQR)	5 (3, 8)
Non-cardiovascular medications count, n (%)	
0–2	1005 (20%)
3–4	1173 (24%)
5–6	1061 (22%)
7+	1673 (34%)
Cardiovascular medications count, median (IQR)	5 (3, 6)
Cardiovascular medications count, n (%)	
0–2	773 (16%)
3–4	1536 (31%)
5–6	1777 (36%)
7+	826 (17%)
Frailty, n (%)	2863 (58%)
Death within 12 months, n (%)	1367 (28%)
Number with ≥1 non-cardiovascular readmission within 12 months, n (%)	1578 (32%)
Number with ≥1 cardiovascular readmission within 12 months, n (%)	935 (19%)
Number of non-cardiovascular readmissions per patient within 12 months, n (%)	
0	3334 (68%)
1	380 (7.7%)
2–3	407 (8.3%)
4+	791 (16%)
Number of cardiovascular readmissions per patient within 12 months, n (%)	
0	3977 (81%)
1	363 (7.4%)
2–3	281 (5.7%)
4+	291 (5.9%)

^1^ Median (Q1, Q3); n (%).

**Table 2:** Factors associated with the cumulative incidence of all-cause readmission within 12 months.

Variable	Subdistribution Hazard Ratio (95% CI)	*p*-Value
Age 55–64 years	1.01 (0.74–1.38)	0.937
Age 65–74 years	1.21 (0.91–1.61)	0.191
Age 75–84 years	**1.37 (1.04–1.80)**	**0.025**
Age 85+ years	**1.43 (1.09–1.87)**	**0.010**
Male Gender	1.09 (0.99–1.20)	0.067
Number of Cardiovascular Conditions in Charlson Comorbidity Index	**1.32 (1.20–1.44)**	**<0.001**
Non-Cardiovascular Polypharmacy	**1.22 (1.11–1.34)**	**<0.001**

Shown in bold are significant associations.

**Table 3:** Factors associated with presence of a non-cardiovascular readmission within 12 months.

Variable	Odds Ratio (95% CI)	*p*-Value
Age 55–64 years	0.91 (0.60–1.37)	0.640
Age 65–74 years	1.08 (0.75–1.58)	0.679
Age 75–84 years	1.17 (0.83–1.69)	0.379
Age 85+ years	1.30 (0.91–1.87)	0.153
Male Gender	1.05 (0.91–1.22)	0.497
Number of Cardiovascular Conditions in Charlson Comorbidity Index	**1.58 (1.36–1.82)**	**<0.001**
Non-Cardiovascular Polypharmacy	**1.48 (1.28–1.72)**	**<0.001**

Shown in bold are significant associations.

## Data Availability

The data that support the findings of this study are available from SA Health, but restrictions apply to the availability of these data, which were used under license for the current study, and so are not publicly available. Data are, however, available from the authors upon reasonable request and with permission of SA Health.

## Supplementary Materials

The following supporting information can be downloaded at: https://www.mdpi.com/article/10.3390/jcm15135275/s1, Supplementary Table S1: Principal Diagnoses for First Non-Cardiovascular Readmissions. Supplementary S1: ICD-10 Codes for Principal Reason for Readmissions. Supplementary S2: ICD-10 Codes for Cardiovascular Conditions in Charlson Comorbidity Index (Excluding Heart Failure). RECORD Checklist.
