# Supplementary material for: Predictors of Non-Cardiovascular Readmissions in Multimorbid Adults with Heart Failure in Australian Hospitals: A Retrospective Cohort Study
Source: J Clin Med. 2026 Jul 6;15(13):5275. doi: 10.3390/jcm15135275 (PMC13363480; doi:10.3390/jcm15135275)
Supplement: Supplementary file 1 [file jcm-15-05275-s001.zip › Supplementary S1.pdf]

Supplementary S1: ICD-10 Codes for Principal Reason for Readmissions

Type	ICD-10 Codes
Non-Cardiovascular Readmission	A010, A020, A021, A028, A029, A038, A039, A041, A042, A043, A044, A045, A047, A048, A049, A050, A059, A064, A071, A072, A078, A080, A081, A082, A083, A084, A085, A090, A099, A150, A151, A152, A153, A156, A157, A159, A160, A161, A162, A180, A182, A183, A184, A188, A190, A199, A279, A280, A2801, A288, A310, A318, A319, A321, A327, A328, A329, A370, A379, A390, A394, A397, A398, A399, A400, A401, A402, A4021, A4022, A403, A408, A409, A410, A411, A412, A413, A414, A4150, A4151, A4152, A4158, A418, A419, A427, A430, A431, A438, A439, A46, A480, A481, A482, A488, A4900, A4901, A491, A4911, A4912, A492, A498, A4981, A4982, A4983, A4984, A4985, A4986, A4987, A4989, A499, A510, A514, A521, A523, A527, A529, A530, A542, A55, A600, A6300, A6301, A6305, A6309, A689, A70, A78, A799, A810, A812, A830, A834, A858, A86, A870, A879, A881, A971, A979, B001, B002, B003, B004, B005, B007, B008, B009, B010, B011, B012, B018, B019, B020, B021, B022, B023, B027, B028, B029, B07, B080, B088, B09, B159, B178, B179, B181, B182, B199, B20, B238, B250, B251, B258, B259, B269, B270, B279, B301, B309, B331, B333, B338, B342, B348, B349, B353, B354, B356, B368, B369, B370, B372, B373, B374, B377, B3781, B3788, B432, B440, B441, B447, B448, B449, B450, B451, B452, B457, B458, B459, B460, B461, B465, B479, B485, B487, B488, B49, B508, B509, B518, B589, B59, B829, B850, B86, B870, B871, B878, B99, C000, C001, C004, C009, C01, C021, C022, C023, C024, C028, C029, C030, C031, C040, C041, C048, C049, C050, C051, C059, C060, C062, C069, C07, C080, C089, C090, C091, C099, C101, C103, C104, C108, C109, C111, C113, C119, C12, C130, C131, C132, C139, C140, C148, C150, C151, C152, C153, C154, C155, C158, C159, C160, C161, C162, C163, C164, C165, C166, C168, C169, C170, C171, C172, C178, C179, C180, C181, C182, C183, C184, C185, C186, C187, C188, C189, C19, C20, C210, C211, C218, C220, C221, C229, C23, C240, C241, C248, C249, C250, C251, C252, C253, C254, C257, C258, C259, C260, C268, C269, C300, C301, C310, C311, C312, C313, C319, C320, C321, C322, C323, C328, C329, C33, C340, C341, C342, C343, C348, C349, C37, C381, C383, C384, C400, C402, C4101, C4102, C411, C412, C413, C414, C418, C419, C431, C432, C433, C434, C435, C436, C437, C439, C440, C441, C442, C443, C444, C445, C446, C447, C448, C449, C450, C451, C452, C457, C459, C460, C467, C471, C480, C481, C482, C488, C490, C491, C492, C493, C494, C495, C496, C499, C500, C501, C502, C503, C504, C505, C506, C508, C509, C511, C518, C519, C52, C530, C539, C541, C548, C549, C55, C56, C570, C574, C577, C578, C58, C600, C601, C602, C608, C609, C61, C621, C629, C632, C638, C639, C64, C65, C66, C670, C671, C672, C673, C674, C675, C676, C678, C679, C680, C690, C693, C694, C696, C697, C699, C700, C701, C710, C711, C712, C713, C714, C715, C716, C717, C718, C719, C720, C729, C73, C740, C741, C749, C753, C755, C760, C761, C762, C763, C7631, C7632, C7639, C765, C767, C770, C771, C772, C773, C774, C775, C778, C779, C780, C781, C782, C783, C784, C785, C786, C787, C788, C790, C791, C792, C793, C794, C795, C796, C797, C7982, C7984, C7988, C799, C800, C809, C810, C811, C812, C814, C817, C819, C820, C821, C822, C823, C827, C829, C830, C831, C833, C837, C838, C839, C840, C841, C844, C845, C846, C847, C848, C849, C851, C852, C857, C859, C860, C861, C862, C863, C864, C865, C866, C8800, C8840, C9000, C9001, C9010, C9011, C9020, C9030, C9100, C9101, C9110, C9111, C9130, C9140, C9150, C9160, C9170, C9180, C9200, C9201, C9210, C9211, C9220, C9230, C9240, C9241, C9250,

	C9251, C9270, C9280, C9281, C9290, C9300, C9310, C9401, C9420, C9430, C9440, C9460, C9461, C9500, C9501, C9510, C9590, C964, C968, C969, D000, D001, D002, D010, D012, D013, D015, D020, D023, D031, D032, D033, D034, D035, D036, D037, D040, D041, D042, D043, D044, D045, D046, D047, D048, D051, D057, D059, D060, D067, D069, D071, D072, D074, D075, D090, D091, D097, D100, D101, D102, D103, D110, D117, D119, D120, D121, D122, D123, D124, D125, D126, D127, D128, D129, D130, D131, D132, D133, D135, D136, D140, D141, D143, D150, D151, D157, D161, D162, D1642, D165, D168, D170, D171, D172, D173, D175, D176, D177, D1801, D1802, D1803, D1804, D1805, D1808, D181, D190, D200, D210, D211, D212, D213, D221, D223, D224, D225, D226, D227, D229, D230, D231, D233, D234, D235, D236, D237, D24, D250, D251, D252, D259, D260, D261, D269, D27, D291, D294, D300, D302, D303, D312, D320, D321, D329, D330, D331, D332, D333, D34, D350, D351, D352, D360, D361, D367, D370, D371, D372, D373, D374, D375, D376, D3771, D3779, D379, D380, D381, D384, D385, D391, D392, D397, D400, D401, D410, D411, D412, D414, D420, D421, D429, D430, D431, D434, D441, D444, D45, D460, D461, D462, D464, D465, D466, D467, D469, D470, D471, D472, D473, D474, D475, D477, D479, D481, D483, D484, D485, D487, D500, D508, D509, D510, D513, D519, D528, D529, D531, D538, D539, D550, D560, D561, D563, D568, D569, D570, D571, D572, D581, D588, D589, D590, D591, D593, D594, D595, D598, D599, D600, D609, D610, D611, D618, D619, D62, D641, D642, D648, D649, D65, D66, D680, D682, D683, D684, D686, D688, D690, D692, D693, D694, D695, D696, D698, D70, D721, D728, D729, D731, D733, D738, D749, D751, D7581, D7589, D759, D761, D763, D800, D801, D803, D806, D808, D821, D839, D841, D848, D849, D860, D861, D862, D863, D868, D869, D890, D891, D892, D893, D898, D8981, D8982, D8989, D899, E02, E032, E035, E038, E039, E041, E042, E048, E049, E050, E052, E055, E058, E059, E060, E061, E063, E064, E069, E078, E079, E099, E1001, E1002, E1011, E1012, E1015, E1016, E1022, E1029, E1031, E1033, E1036, E1040, E1041, E1042, E1043, E1051, E1052, E1061, E1064, E1065, E1069, E1073, E109, E1101, E1102, E1111, E1112, E1113, E1114, E1115, E1116, E1121, E1122, E1129, E1131, E1133, E1134, E1135, E1136, E1139, E1140, E1141, E1142, E1143, E1151, E1152, E1161, E1162, E1163, E1164, E1165, E1169, E1171, E1172, E1173, E119, E1301, E1311, E1315, E1322, E1329, E1342, E1352, E1364, E1365, E1373, E139, E1401, E1411, E1413, E1429, E1439, E1440, E1442, E1451, E1452, E1464, E1465, E1472, E1473, E149, E15, E160, E161, E162, E209, E210, E211, E212, E213, E222, E230, E231, E232, E236, E242, E248, E249, E250, E260, E261, E269, E271, E272, E273, E274, E278, E279, E340, E349, E40, E43, E440, E46, E512, E519, E52, E538, E559, E58, E611, E612, E65, E662, E6620, E6623, E6690, E6693, E672, E673, E713, E722, E738, E739, E740, E743, E752, E781, E782, E785, E802, E804, E806, E831, E833, E834, E835, E84, E850, E853, E854, E858, E859, E86, E870, E871, E872, E873, E875, E876, E877, E878, E880, E882, E883, E888, E892, E893, E898, F000, F0000, F0001, F001, F0010, F0011, F002, F0020, F0021, F009, F0090, F0091, F010, F0101, F011, F0110, F0111, F0120, F0121, F013, F0130, F0131, F018, F0180, F0181, F019, F0190, F0191, F020, F0200, F0201, F0221, F023, F0230, F0231, F028, F0280, F0281, F03, F0300, F0301, F0401, F0402, F0403, F049, F050, F051, F058, F059, F061, F062, F0630, F0631, F0633, F0639, F064, F065, F067, F068, F069, F070, F072, F078, F09, F100, F101, F102, F103, F104, F105, F106, F107, F108, F110, F111, F112, F113, F114, F115, F118, F120, F121, F123, F124, F125, F128, F1300, F1301, F1309, F1311, F1319, F1320, F1321, F1329,

	F1330, F1331, F1339, F1341, F1349, F1350, F1351, F1359, F143, F145, F1500, F1501, F1509, F1510, F1511, F1519, F1521, F1531, F1541, F1550, F1551, F1552, F1559, F1571, F1579, F1581, F1591, F1599, F1650, F1659, F190, F191, F192, F193, F195, F197, F198, F199, F200, F201, F202, F203, F205, F208, F209, F21, F220, F228, F229, F2300, F2310, F2320, F2321, F2330, F2331, F2380, F2381, F2390, F2391, F250, F251, F252, F258, F259, F28, F29, F300, F301, F302, F309, F310, F311, F312, F313, F314, F315, F316, F317, F318, F319, F3200, F3210, F3220, F3221, F3230, F3231, F3280, F3290, F3291, F330, F331, F332, F333, F338, F339, F340, F341, F348, F381, F388, F39, F4000, F4001, F402, F410, F411, F412, F413, F418, F419, F421, F422, F428, F429, F430, F431, F432, F438, F439, F440, F441, F444, F445, F446, F447, F4480, F4488, F449, F450, F452, F4532, F4534, F454, F458, F459, F500, F501, F505, F509, F511, F522, F552, F59, F600, F601, F602, F6030, F6031, F604, F605, F606, F607, F608, F609, F61, F630, F658, F681, F69, F709, F719, F721, F728, F729, F780, F788, F791, F798, F799, F84, F900, F911, F918, F919, F920, F928, F929, F952, F958, F988, F99, G000, G001, G002, G009, G01, G021, G030, G031, G038, G039, G040, G042, G048, G049, G050, G051, G052, G060, G061, G062, G08, G10, G111, G112, G114, G119, G122, G130, G14, G20, G210, G211, G214, G22, G231, G232, G233, G239, G240, G244, G248, G249, G250, G251, G252, G253, G254, G255, G258, G259, G300, G301, G308, G309, G310, G311, G312, G313, G318, G319, G320, G35, G360, G371, G372, G373, G378, G379, G4000, G4001, G4010, G4011, G4020, G4021, G4030, G4031, G4040, G4041, G4050, G4060, G4061, G4070, G4080, G4081, G4090, G4091, G410, G411, G412, G418, G419, G430, G431, G432, G433, G438, G439, G440, G442, G443, G444, G448, G450, G451, G453, G454, G458, G459, G460, G465, G467, G470, G471, G472, G4731, G4732, G4733, G474, G478, G479, G500, G501, G508, G510, G518, G519, G521, G522, G527, G530, G540, G541, G544, G546, G548, G549, G550, G551, G552, G553, G560, G561, G562, G563, G569, G570, G571, G572, G573, G575, G576, G578, G579, G580, G5810, G5811, G5812, G5819, G587, G588, G600, G603, G608, G609, G610, G618, G620, G621, G628, G629, G630, G631, G635, G636, G638, G700, G708, G709, G710, G711, G713, G718, G720, G721, G723, G724, G728, G729, G731, G8000, G804, G809, G811, G819, G8200, G8210, G8212, G8213, G8214, G8216, G8221, G8224, G8226, G8242, G8243, G8244, G8246, G8255, G8256, G830, G831, G832, G833, G834, G836, G8381, G8389, G839, G904, G9062, G9070, G9072, G9079, G908, G909, G910, G911, G912, G913, G918, G919, G92, G920, G928, G929, G930, G931, G932, G933, G934, G935, G936, G938, G939, G941, G943, G950, G951, G952, G958, G959, G960, G961, G968, G969, G970, G9711, G972, G9731, G9734, G978, G979, G98, G990, G992, H000, H010, H019, H020, H021, H022, H023, H024, H025, H028, H029, H031, H040, H041, H042, H043, H044, H045, H048, H050, H051, H053, H058, H062, H101, H103, H104, H108, H109, H110, H113, H114, H118, H119, H131, H133, H150, H151, H158, H160, H161, H162, H163, H168, H169, H178, H179, H181, H182, H184, H185, H186, H187, H188, H189, H191, H192, H193, H200, H201, H208, H209, H210, H211, H214, H220, H221, H250, H251, H252, H258, H259, H261, H262, H268, H269, H270, H271, H278, H279, H308, H309, H313, H314, H318, H330, H332, H333, H334, H335, H341, H342, H348, H349, H350, H352, H353, H357, H358, H359, H400, H401, H402, H403, H404, H405, H406, H408, H409, H430, H431, H433, H438, H439, H440, H441, H444, H445, H447, H449, H46, H470, H471, H472, H473, H481, H490, H491, H492, H498, H499, H500, H505, H508, H509, H512, H522, H526, H531, H532, H533, H534, H538, H539, H541, H542, H544, H545, H549, H55, H571, H578,

	H579, H588, H590, H5913, H5915, H5919, H5982, H5983, H5985, H5989, H600, H601, H602, H603, H604, H605, H608, H609, H610, H611, H612, H613, H622, H651, H654, H659, H660, H663, H664, H669, H699, H700, H701, H702, H708, H709, H71, H728, H729, H738, H740, H744, H748, H749, H801, H810, H811, H812, H813, H814, H818, H819, H830, H832, H833, H838, H900, H903, H904, H905, H918, H919, H920, H921, H931, H933, H939, H958, I00, I269, I7300, I7301, I774, I776, I778, I779, I780, I781, I788, I789, I800, I801, I802, I8020, I8021, I8022, I8023, I803, I8040, I8041, I8042, I808, I81, I820, I850, I859, I861, I862, I864, I868, I870, I871, I872, I878, I879, I880, I888, I890, I891, I898, I950, I951, I9510, I9511, I9512, I9519, I952, I958, I959, I970, I972, I9734, I9735, I978, I9781, I9782, I9783, I9789, I979, I982, I983, I99, J00, J010, J011, J013, J014, J018, J019, J020, J028, J029, J038, J039, J040, J041, J050, J051, J060, J069, J09, J100, J101, J108, J110, J111, J120, J121, J122, J123, J128, J129, J13, J14, J150, J151, J152, J153, J154, J155, J156, J157, J158, J159, J161, J168, J170, J171, J172, J173, J178, J180, J181, J182, J188, J189, J201, J204, J205, J206, J208, J209, J210, J211, J219, J22, J300, J303, J310, J311, J312, J320, J321, J322, J323, J324, J328, J329, J330, J338, J339, J340, J341, J342, J343, J348, J350, J351, J353, J358, J359, J36, J370, J3800, J3801, J3803, J3804, J381, J382, J383, J384, J385, J386, J387, J390, J391, J392, J398, J40, J42, J439, J440, J441, J448, J449, J450, J451, J459, J46, J47, J60, J61, J628, J634, J662, J672, J678, J679, J680, J690, J698, J700, J701, J702, J703, J704, J708, J80, J82, J841, J848, J849, J850, J851, J852, J860, J869, J90, J920, J929, J930, J931, J938, J939, J940, J942, J948, J949, J9501, J9502, J9503, J9504, J9509, J955, J9564, J9569, J958, J9581, J9582, J9584, J9585, J9589, J9600, J9601, J9609, J9610, J9611, J9619, J9690, J9691, J9699, J980, J981, J982, J984, J985, J986, J987, J988, J989, J991, J998, K011, K025, K028, K029, K046, K047, K048, K049, K051, K052, K053, K056, K068, K073, K074, K076, K083, K0888, K089, K092, K098, K102, K103, K108, K109, K112, K113, K115, K116, K117, K118, K119, K120, K121, K122, K123, K130, K132, K134, K137, K140, K146, K148, K149, K20, K210, K219, K220, K221, K222, K223, K224, K225, K226, K227, K228, K229, K250, K251, K252, K253, K254, K255, K256, K257, K259, K260, K261, K262, K263, K264, K265, K266, K267, K269, K270, K273, K274, K275, K277, K279, K281, K284, K285, K287, K289, K290, K291, K2920, K2921, K2930, K2941, K2950, K2951, K2960, K2961, K2970, K2971, K2980, K2981, K30, K311, K314, K315, K316, K317, K3181, K3182, K3188, K319, K352, K353, K358, K36, K37, K381, K388, K4000, K4001, K4020, K4021, K4030, K4031, K4040, K4041, K4090, K4091, K412, K413, K414, K419, K420, K421, K429, K430, K431, K432, K433, K434, K435, K436, K437, K439, K440, K449, K450, K451, K458, K460, K469, K500, K501, K508, K509, K510, K512, K513, K514, K515, K518, K519, K520, K521, K522, K523, K528, K529, K5521, K5522, K5531, K5532, K558, K560, K561, K562, K563, K564, K565, K566, K567, K5700, K5701, K5702, K5703, K5710, K5711, K5712, K5713, K5720, K5721, K5722, K5723, K5730, K5731, K5732, K5733, K5742, K5750, K5751, K5753, K5780, K5782, K5790, K5791, K5792, K5793, K580, K581, K582, K588, K589, K590, K591, K592, K593, K594, K598, K599, K601, K602, K603, K605, K610, K611, K612, K613, K614, K620, K621, K623, K624, K625, K626, K627, K628, K629, K630, K631, K632, K633, K634, K6350, K6358, K638, K639, K640, K641, K642, K643, K644, K645, K648, K649, K650, K6511, K6522, K6529, K653, K658, K659, K660, K661, K662, K668, K669, K700, K701, K702, K703, K704, K709, K710, K711, K712, K716, K717, K718, K719, K720, K721, K729, K732, K739, K740, K743, K744, K746, K750, K751, K754, K758, K759, K760, K761, K764, K766, K767, K768, K769, K778, K8000, K8001, K8010, K8011,

	K8020, K8021, K8030, K8031, K8040, K8041, K8050, K8051, K8080, K8081, K810, K811, K818, K819, K821, K822, K823, K824, K828, K829, K830, K831, K833, K834, K838, K839, K850, K851, K852, K853, K858, K859, K860, K861, K862, K863, K868, K869, K900, K904, K908, K909, K911, K912, K913, K914, K9141, K9142, K9143, K9149, K9161, K9162, K9163, K9164, K9166, K9167, K9169, K918, K9181, K9182, K9183, K9184, K9189, K919, K920, K921, K922, K928, K929, L010, L011, L020, L021, L022, L023, L0241, L0242, L0243, L028, L029, L0301, L0302, L0312, L0313, L0314, L0319, L032, L033, L038, L039, L040, L042, L050, L059, L080, L088, L089, L100, L102, L108, L109, L111, L120, L121, L129, L130, L131, L138, L139, L208, L209, L219, L22, L230, L235, L238, L239, L244, L245, L248, L249, L251, L253, L258, L259, L26, L270, L271, L278, L280, L281, L282, L298, L299, L301, L302, L303, L304, L308, L309, L400, L401, L403, L405, L408, L409, L411, L432, L439, L440, L500, L508, L509, L511, L512, L518, L519, L52, L530, L538, L539, L550, L551, L561, L568, L570, L580, L589, L598, L599, L600, L608, L609, L700, L710, L711, L720, L721, L728, L729, L732, L738, L739, L740, L812, L814, L817, L818, L819, L82, L83, L840, L851, L853, L858, L859, L88, L8904, L8905, L8906, L8910, L8912, L8914, L8915, L8916, L8917, L8919, L8920, L8921, L8924, L8925, L8926, L8927, L8929, L8934, L8935, L8936, L8937, L8939, L8942, L8944, L8945, L8946, L8947, L8949, L8954, L8956, L8957, L8958, L8959, L8990, L8992, L8993, L8994, L8995, L8996, L8997, L8998, L8999, L900, L9050, L9051, L9059, L9100, L9101, L9109, L918, L923, L928, L929, L931, L932, L940, L942, L944, L950, L958, L959, L970, L978, L979, L980, L981, L982, L983, L984, L987, L988, L989, L990, L998, M0001, M0002, M0003, M0004, M0005, M0006, M0007, M0008, M0009, M0020, M0021, M0022, M0023, M0024, M0025, M0026, M0027, M0028, M0081, M0082, M0083, M0084, M0085, M0086, M0087, M0088, M0091, M0092, M0093, M0094, M0095, M0096, M0097, M0098, M0116, M0133, M0136, M0156, M0166, M0284, M0286, M0287, M0293, M0294, M0296, M0297, M0509, M0514, M0519, M0520, M0528, M0584, M0590, M0591, M0592, M0593, M0594, M0596, M0597, M0598, M0599, M0600, M0601, M0603, M0606, M0607, M0608, M0609, M0610, M0611, M0619, M0632, M0633, M0634, M0637, M0640, M0641, M0642, M0643, M0644, M0645, M0646, M0647, M0681, M0682, M0683, M0684, M0685, M0687, M0690, M0691, M0692, M0693, M0694, M0695, M0696, M0697, M0698, M0699, M0737, M0738, M0746, M1000, M1002, M1003, M1004, M1005, M1006, M1007, M1009, M1023, M1026, M1027, M1033, M1037, M1090, M1091, M1092, M1093, M1094, M1095, M1096, M1097, M1098, M1099, M1114, M1120, M1121, M1122, M1123, M1124, M1125, M1126, M1127, M1128, M1129, M1180, M1181, M1182, M1186, M1192, M1194, M1196, M1197, M1231, M1253, M1254, M1256, M1286, M1288, M130, M1313, M1314, M1316, M1317, M1380, M1381, M1382, M1383, M1384, M1385, M1386, M1387, M1388, M1389, M1390, M1391, M1392, M1393, M1394, M1395, M1396, M1397, M1398, M1399, M146, M150, M151, M152, M154, M160, M161, M162, M163, M164, M165, M167, M170, M171, M173, M175, M181, M1901, M1902, M1903, M1904, M1907, M1908, M1909, M1911, M1913, M1917, M1923, M1981, M1983, M1984, M1987, M1988, M200, M201, M202, M204, M205, M2106, M2116, M2117, M2124, M2133, M2137, M214, M2157, M2167, M2176, M2186, M2191, M2194, M2196, M220, M221, M222, M2320, M2322, M2323, M2325, M2326, M2329, M2333, M2339, M2344, M2349, M2351, M2352, M2359, M2381, M2389, M2390, M2421, M2427, M2428, M2431, M2434, M2435, M2436, M2441, M2442, M2444, M2445, M2446, M2447, M2449,

	M2452, M2454, M2457, M2466, M2481, M2494, M2501, M2502, M2503, M2505, M2506, M2507, M2515, M2522, M2531, M2533, M2534, M2537, M2538, M2541, M2542, M2543, M2545, M2546, M2547, M2549, M2550, M2551, M2552, M2553, M2554, M2555, M2556, M2557, M2558, M2559, M2561, M2562, M2564, M2566, M2567, M2568, M2575, M2578, M2584, M2591, M2593, M2595, M2596, M2597, M2598, M300, M301, M308, M310, M311, M313, M314, M315, M316, M317, M318, M319, M320, M321, M328, M329, M331, M332, M341, M348, M349, M350, M351, M352, M353, M354, M358, M359, M4022, M4023, M4024, M4194, M4196, M4307, M4312, M4316, M4317, M436, M4502, M4506, M4507, M4509, M461, M4620, M4622, M4623, M4624, M4625, M4626, M4627, M4628, M4632, M4634, M4636, M4637, M4644, M4646, M4647, M4652, M4654, M4656, M4657, M4682, M4687, M4692, M4696, M4697, M4706, M4712, M4714, M4716, M4717, M4722, M4723, M4726, M4727, M4780, M4782, M4783, M4784, M4786, M4787, M4792, M4793, M4794, M4795, M4796, M4797, M4798, M4799, M4800, M4802, M4803, M4804, M4805, M4806, M4807, M4808, M4809, M4812, M4842, M4844, M4845, M4846, M4848, M4850, M4854, M4855, M4856, M4857, M4882, M4899, M4906, M4952, M4954, M4955, M4956, M4957, M4958, M500, M501, M502, M503, M509, M510, M511, M512, M513, M519, M5327, M533, M5382, M5386, M5387, M5397, M5411, M5412, M5414, M5415, M5416, M5417, M5418, M5419, M542, M543, M544, M545, M546, M5480, M5481, M5483, M5484, M5485, M5486, M5487, M5488, M5489, M5492, M5494, M5495, M5496, M5497, M5498, M5499, M6001, M6002, M6003, M6005, M6008, M6009, M6080, M6081, M6082, M6083, M6085, M6086, M6087, M6088, M6089, M6090, M6091, M6094, M6095, M6096, M6098, M6099, M6155, M6215, M6218, M6239, M6246, M6250, M6251, M6252, M6253, M6255, M6256, M6258, M6259, M6262, M6265, M6266, M6268, M6269, M6280, M6282, M6285, M6286, M6288, M6289, M6295, M6298, M6504, M6513, M6514, M6517, M6523, M653, M654, M6581, M6583, M6584, M6585, M6586, M6591, M6593, M6594, M6595, M6596, M6597, M6598, M660, M6618, M6624, M6641, M6645, M6646, M6647, M6655, M670, M6716, M6743, M6744, M6746, M6749, M6783, M6784, M6785, M6787, M6794, M6795, M700, M702, M703, M704, M705, M706, M707, M7103, M7104, M7106, M7112, M7113, M7115, M7116, M7117, M712, M7138, M7155, M7195, M720, M722, M7244, M7260, M7262, M7263, M7264, M7265, M7266, M7267, M7268, M7269, M7295, M7296, M750, M751, M753, M754, M755, M758, M760, M763, M766, M767, M768, M773, M774, M775, M7910, M7911, M7913, M7914, M7915, M7916, M7918, M7919, M7924, M7925, M7926, M7927, M7928, M7930, M7932, M7938, M7939, M7954, M7956, M7957, M7958, M7962, M7963, M7964, M7965, M7966, M7967, M7970, M7971, M7975, M7976, M7977, M7978, M7979, M7981, M7982, M7983, M7984, M7985, M7986, M7987, M7988, M7991, M7992, M7995, M7998, M8008, M8025, M8028, M8043, M8045, M8048, M8053, M8058, M8083, M8085, M8086, M8087, M8088, M8090, M8091, M8092, M8093, M8094, M8095, M8096, M8097, M8098, M8099, M8148, M8185, M8188, M8189, M8195, M8198, M8199, M8386, M8401, M8402, M8403, M8404, M8405, M8406, M8407, M8408, M8411, M8412, M8413, M8414, M8415, M8416, M8417, M8418, M8419, M8421, M8422, M8425, M8426, M8435, M8436, M8437, M8440, M8441, M8442, M8445, M8446, M8447, M8448, M8558, M8566, M8584, M8585, M8587, M8588, M8595, M8607, M8611, M8612, M8614, M8615, M8616, M8617, M8618, M8627, M8628, M8636, M8637, M8641, M8642, M8644,

	M8645, M8647, M8662, M8663, M8664, M8665, M8666, M8667, M8668, M8684, M8685, M8686, M8687, M8688, M8690, M8691, M8692, M8693, M8694, M8695, M8696, M8697, M8698, M8699, M8705, M8707, M8715, M8716, M8718, M8725, M8735, M8738, M8784, M8785, M8787, M8788, M8794, M8795, M8796, M8797, M8885, M8888, M889, M8952, M8954, M8980, M8981, M8985, M8987, M8988, M8995, M8998, M8999, M9070, M9071, M9072, M9073, M9075, M9076, M9078, M9079, M9326, M940, M941, M9435, M9485, M9488, M950, M952, M954, M960, M961, M966, M9679, M968, N005, N007, N009, N017, N018, N019, N025, N027, N028, N029, N031, N032, N038, N040, N041, N042, N043, N045, N047, N048, N049, N050, N052, N055, N057, N058, N059, N082, N085, N10, N110, N111, N118, N119, N12, N130, N131, N132, N133, N134, N135, N136, N137, N138, N139, N140, N141, N142, N151, N158, N159, N160, N164, N170, N178, N179, N181, N183, N184, N185, N189, N19, N200, N201, N202, N209, N210, N211, N218, N23, N250, N251, N258, N259, N26, N281, N288, N289, N298, N300, N301, N302, N304, N308, N309, N312, N318, N319, N320, N321, N322, N324, N328, N329, N340, N341, N342, N350, N358, N359, N360, N362, N368, N369, N390, N393, N3930, N3931, N394, N3981, N3988, N399, N40, N410, N411, N412, N413, N418, N419, N421, N428, N429, N431, N432, N433, N434, N450, N459, N47, N480, N481, N482, N483, N484, N485, N488, N489, N491, N492, N498, N501, N508, N511, N512, N518, N600, N602, N604, N608, N61, N62, N63, N641, N644, N645, N648, N649, N701, N709, N719, N72, N730, N735, N736, N738, N739, N743, N750, N751, N760, N761, N762, N763, N764, N765, N766, N768, N771, N800, N801, N803, N805, N810, N811, N812, N813, N814, N815, N816, N818, N819, N820, N823, N824, N825, N829, N830, N832, N835, N838, N839, N840, N841, N842, N850, N851, N858, N859, N871, N879, N893, N898, N899, N903, N904, N906, N907, N908, N912, N920, N921, N923, N924, N925, N926, N938, N939, N946, N948, N949, N950, N951, N952, N958, N991, N992, N993, N994, N9951, N9952, N9953, N9959, N9962, N9963, N9964, N9969, N998, N9981, N9982, N9983, N9989, N999, O049, O80, Q010, Q0189, Q0301, Q031, Q0389, Q039, Q0461, Q0520, Q0540, Q0590, Q069, Q070, Q180, Q181, Q206, Q310, Q311, Q313, Q320, Q387, Q396, Q401, Q430, Q4331, Q4339, Q4389, Q504, Q541, Q612, Q613, Q619, Q6217, Q6239, Q6309, Q6589, Q6600, Q667, Q760, Q772, Q790, Q796, Q822, Q8289, Q859, Q8781, Q8923, Q8924, Q992, R002, R008, R02, R031, R040, R041, R042, R048, R05, R060, R061, R062, R064, R066, R068, R070, R071, R072, R073, R074, R090, R091, R092, R093, R0988, R0989, R100, R101, R102, R103, R104, R11, R12, R13, R14, R15, R160, R161, R162, R17, R170, R18, R190, R194, R195, R198, R1989, R202, R203, R208, R21, R220, R221, R222, R224, R229, R231, R232, R233, R234, R238, R250, R251, R252, R253, R258, R260, R261, R262, R263, R268, R270, R278, R291, R295, R296, R2988, R2989, R300, R309, R31, R32, R33, R34, R35, R36, R390, R391, R392, R398, R400, R401, R402, R410, R413, R418, R42, R440, R441, R442, R443, R448, R451, R452, R454, R456, R4581, R4589, R462, R464, R468, R470, R471, R478, R482, R490, R498, R502, R508, R509, R51, R520, R522, R529, R53, R54, R55, R568, R571, R579, R58, R590, R591, R599, R600, R601, R609, R619, R630, R631, R633, R634, R638, R64, R680, R682, R688, R69, R701, R71, R72, R73, R740, R748, R768, R770, R778, R779, R790, R798, R7982, R7983, R7989, R799, R80, R827, R836, R838, R845, R855, R868, R875, R876, R878, R895, R900, R908, R91, R930, R932, R933, R934, R9351, R9359, R937, R938, R944, R945, S0000, S0001, S0005, S0008, S001, S0020, S0021, S0023, S0028, S0030, S0031, S0034, S0035, S0038, S0041, S0045, S0048, S0051, S0055, S0058,

	S0080, S0081, S0083, S0085, S0088, S0090, S0091, S0095, S0098, S010, S011, S0120, S0121, S0122, S0123, S0129, S0130, S0131, S0133, S0134, S0139, S0141, S0143, S0149, S0151, S0154, S0159, S017, S0188, S019, S020, S021, S022, S023, S024, S025, S0260, S0261, S0262, S0263, S0264, S0265, S0266, S0268, S0269, S027, S028, S029, S030, S034, S045, S050, S051, S052, S053, S054, S055, S056, S058, S059, S0600, S0601, S0602, S0603, S0604, S061, S0620, S0621, S0622, S0623, S0628, S0630, S0631, S0632, S0633, S0634, S0638, S068, S069, S080, S081, S090, S092, S098, S099, S100, S1011, S1085, S1090, S1091, S1095, S1098, S111, S117, S1188, S119, S120, S121, S1221, S1222, S1223, S1224, S1225, S128, S130, S1311, S1315, S1316, S134, S136, S140, S1410, S1411, S1412, S1413, S142, S143, S1501, S1503, S151, S152, S153, S158, S159, S16, S198, S199, S200, S2012, S202, S2030, S2031, S2038, S2040, S2041, S2048, S2080, S2081, S210, S211, S212, S219, S2200, S2201, S2202, S2203, S2204, S2205, S2206, S222, S2231, S2232, S2240, S2241, S2242, S2243, S2244, S225, S2315, S233, S234, S235, S240, S2410, S2411, S2412, S250, S251, S255, S258, S260, S2681, S2682, S2688, S270, S271, S272, S2731, S2732, S2738, S276, S2781, S2783, S2788, S290, S298, S299, S300, S301, S302, S3021, S3080, S3081, S3084, S3088, S3090, S3091, S310, S311, S312, S313, S314, S3180, S3200, S3201, S3202, S3203, S3204, S3205, S321, S322, S323, S324, S325, S3281, S3282, S3283, S3289, S3311, S3313, S3314, S333, S3351, S337, S341, S342, S343, S352, S354, S355, S357, S358, S359, S3600, S3601, S3602, S3603, S3604, S3608, S3610, S3611, S3612, S3613, S3614, S3615, S3620, S3621, S363, S3640, S3641, S3649, S3650, S3651, S3652, S3653, S3654, S3659, S366, S3681, S3682, S3683, S3688, S369, S3700, S3701, S3702, S371, S3720, S3721, S3722, S3730, S3732, S3738, S3781, S381, S390, S398, S399, S400, S4081, S409, S410, S411, S417, S4180, S4200, S4201, S4202, S4203, S4210, S4211, S4212, S4213, S4214, S4219, S4220, S4221, S4222, S4223, S4224, S4229, S423, S4240, S4241, S4242, S4243, S4244, S4245, S4249, S427, S428, S429, S4300, S4301, S4302, S4303, S4308, S431, S434, S435, S437, S440, S441, S442, S449, S451, S453, S458, S459, S460, S461, S462, S463, S468, S469, S498, S499, S500, S501, S507, S5081, S5082, S5088, S509, S510, S517, S5188, S519, S5200, S5201, S5202, S5209, S5210, S5211, S5212, S5220, S5221, S5230, S5231, S524, S5250, S5251, S5252, S5253, S5259, S526, S527, S528, S530, S5310, S5312, S5314, S5318, S533, S5340, S540, S541, S542, S543, S550, S551, S552, S561, S562, S563, S564, S565, S568, S598, S599, S600, S601, S602, S6081, S6082, S6083, S6084, S6088, S609, S610, S611, S617, S6188, S619, S620, S6211, S6212, S6214, S6216, S6217, S6219, S6220, S6221, S6222, S6224, S6230, S6231, S6232, S6233, S6234, S624, S6250, S6251, S6252, S6260, S6261, S6262, S6263, S627, S628, S6308, S6310, S6311, S6312, S634, S6350, S6360, S6361, S637, S640, S641, S642, S643, S644, S648, S649, S650, S651, S654, S655, S658, S659, S660, S661, S662, S663, S666, S668, S669, S670, S680, S681, S682, S684, S698, S699, S700, S701, S7081, S7088, S709, S710, S711, S7200, S7201, S7202, S7203, S7204, S7205, S7208, S7210, S7211, S722, S723, S7240, S7241, S7243, S7244, S727, S728, S729, S7300, S7301, S7302, S7308, S7310, S7318, S740, S750, S759, S760, S761, S762, S763, S764, S767, S798, S799, S800, S801, S8081, S8082, S8083, S8088, S809, S810, S817, S8188, S819, S820, S8211, S8218, S8221, S8228, S8231, S8238, S8240, S8241, S8242, S8249, S825, S826, S827, S8281, S8282, S8288, S829, S830, S8310, S8311, S8312, S8314, S832, S8341, S8342, S8343, S8344, S8350, S8352, S8353, S8354, S836, S841, S848, S849, S850,

	S851, S853, S858, S859, S860, S861, S862, S863, S868, S869, S878, S881, S898, S899, S900, S903, S9082, S9084, S9088, S909, S910, S911, S912, S913, S917, S920, S921, S9221, S9222, S9223, S923, S924, S925, S930, S9311, S9312, S9332, S9333, S9338, S9340, S9341, S9343, S9348, S936, S942, S949, S950, S959, S961, S968, S969, S978, S981, S982, S998, S999, T0904, T093, T1105, T111, T116, T119, T1305, T131, T139, T158, T16, T171, T172, T173, T174, T175, T178, T179, T180, T181, T182, T183, T184, T185, T188, T189, T190, T191, T198, T200, T201, T202, T203, T2101, T2102, T2103, T2104, T2105, T2114, T2120, T2121, T2122, T2123, T2124, T2125, T2130, T2131, T2132, T2133, T2134, T2139, T2201, T2212, T2220, T2221, T2222, T2230, T2231, T2232, T230, T231, T232, T233, T240, T241, T242, T243, T250, T251, T252, T253, T270, T272, T273, T280, T281, T291, T292, T361, T364, T375, T378, T380, T381, T383, T385, T388, T390, T391, T393, T401, T402, T403, T404, T406, T407, T4121, T4122, T420, T421, T423, T424, T426, T427, T428, T430, T431, T432, T433, T435, T4361, T4362, T4369, T440, T443, T446, T447, T450, T451, T455, T460, T461, T462, T463, T464, T465, T466, T467, T471, T472, T474, T484, T485, T490, T495, T497, T500, T501, T502, T503, T504, T506, T509, T510, T518, T519, T528, T537, T542, T549, T560, T561, T568, T58, T594, T598, T599, T603, T604, T608, T620, T622, T628, T629, T630, T633, T634, T652, T658, T659, T670, T671, T673, T675, T676, T678, T68, T690, T700, T703, T704, T709, T71, T730, T740, T741, T748, T751, T780, T781, T782, T783, T784, T792, T793, T794, T796, T797, T798, T799, T801, T802, T806, T808, T809, T810, T811, T812, T813, T814, T815, T817, T818, T8182, T8183, T8184, T8189, T819, T830, T831, T832, T833, T8341, T835, T836, T8361, T8362, T838, T8381, T8383, T8384, T8385, T8389, T840, T841, T842, T843, T844, T845, T846, T847, T848, T8481, T8482, T8483, T8485, T8489, T849, T850, T852, T853, T854, T855, T856, T8561, T8562, T8563, T8564, T8565, T8569, T8571, T8572, T8573, T8574, T8575, T8576, T8577, T8578, T8581, T8582, T8583, T8584, T8585, T8586, T8588, T859, T860, T861, T864, T865, T8681, T8685, T8686, T8689, T870, T872, T873, T874, T875, T876, T8761, T8762, T8769, T880, T881, T8859, T886, T887, T888, U0711, U0712, U072, Z004, Z006, Z008, Z015, Z016, Z018, Z029, Z031, Z032, Z036, Z038, Z0381, Z0389, Z039, Z041, Z043, Z044, Z045, Z048, Z049, Z080, Z081, Z082, Z087, Z088, Z089, Z090, Z092, Z094, Z097, Z098, Z099, Z115, Z121, Z126, Z128, Z131, Z136, Z1381, Z1383, Z139, Z208, Z227, Z258, Z268, Z269, Z291, Z292, Z2929, Z298, Z301, Z3021, Z305, Z4000, Z4001, Z4008, Z408, Z409, Z411, Z4181, Z4182, Z4189, Z419, Z420, Z421, Z422, Z423, Z424, Z428, Z430, Z431, Z432, Z433, Z434, Z435, Z436, Z438, Z441, Z443, Z448, Z451, Z452, Z4581, Z4582, Z4583, Z4589, Z462, Z463, Z465, Z466, Z467, Z468, Z470, Z478, Z480, Z488, Z489, Z49.1, Z490, Z491, Z492, Z509, Z510, Z511, Z513, Z514, Z5161, Z5169, Z5181, Z5188, Z519, Z524, Z526, Z527, Z528, Z540, Z542, Z544, Z547, Z548, Z590, Z591, Z597, Z598, Z602, Z608, Z609, Z630, Z711, Z713, Z718, Z719, Z733, Z738, Z739, Z740, Z741, Z742, Z743, Z748, Z750, Z7510, Z7511, Z7512, Z7513, Z7514, Z7518, Z7519, Z752, Z753, Z7540, Z7541, Z7549, Z755, Z758, Z759, Z765, Z768, Z769, Z800
Cardiovascular Readmissions	D735, G463, G464, I011, I050, I051, I052, I058, I059, I061, I062, I071, I078, I080, I081, I082, I083, I088, I099, I10, I110, I119, I120, I129, I130, I131, I132, I139, I150, I151, I152, I158, I159, I200, I201, I208, I209, I210, I211, I212, I213, I214, I219, I220, I221, I229, I232, I233, I238, I240, I241, I248, I249, I250, I2510, I2511, I2512, I2513, I252, I253, I254, I255, I256, I258, I259, I260, I270, I272, I279, I288, I300, I301, I308, I309, I311, I312, I313, I318, I319, I328, I330, I339, I340, I341, I348, I350, I351, I352, I358, I359, I371, I378, I38, I398, I400, I401, I408, I409, I418, I420, I421, I422, I423, I424, I425, I426, I427, I428, I429, I430,

	I431, I438, I440, I441, I442, I443, I447, I451, I452, I453, I454, I455, I456, I458, I459, I460, I461, I469, I470, I471, I472, I479, I480, I481, I482, I483, I484, I489, I490, I491, I492, I493, I494, I495, I4950, I4951, I498, I499, I500, I501, I509, I510, I513, I514, I517, I518, I519, I600, I601, I602, I603, I604, I605, I606, I607, I608, I609, I610, I611, I612, I613, I614, I615, I616, I618, I619, I620, I621, I629, I630, I631, I632, I633, I634, I635, I636, I638, I639, I64, I650, I651, I652, I653, I658, I659, I660, I661, I662, I663, I664, I668, I669, I670, I671, I672, I673, I674, I676, I677, I678, I679, I680, I700, I701, I7020, I7021, I7022, I7023, I7024, I708, I709, I7100, I7101, I7102, I7103, I711, I712, I713, I714, I715, I716, I719, I720, I721, I722, I723, I724, I725, I726, I728, I729, I731, I738, I739, I740, I741, I742, I743, I744, I745, I748, I749, I770, I771, I772, I773, I822, I823, I828, I830, I831, I832, I839, J81, K550, K551, K559, K763, M6222, M6223, M6224, M6225, M6226, N280, Q2110, Q2111, Q2112, Q2119, Q2120, Q2383, Q245, Q2519, Q2530, Q2549, Q2571, Q273, Q279, Q280, Q281, Q282, Q2839, R000, R001, R003, R030, R570, R931, R943, S064, S065, S066, T820, T821, T822, T823, T824, T8251, T8252, T8253, T8259, T826, T827, T8271, T8272, T8273, T8274, T8275, T8276, T8277, T8279, T828, T8281, T8282, T8283, T8284, T8285, T8286, T8289, T829, T862, Z035, Z450
