# Supplementary material for: Predictors of Non-Cardiovascular Readmissions in Multimorbid Adults with Heart Failure in Australian Hospitals: A Retrospective Cohort Study
Source: J Clin Med. 2026 Jul 6;15(13):5275. doi: 10.3390/jcm15135275 (PMC13363480; doi:10.3390/jcm15135275)
Supplement: Supplementary file 1 [file jcm-15-05275-s001.zip › Supplementary S2.pdf]

Supplementary S2: ICD-10 Codes for Cardiovascular Conditions in Charlson Comorbidity Index (Excluding Heart Failure)

Type	ICD-10 Codes
Acute myocardial infarction	I21.0, I21.1, I21.2, I21.3, I21.4, I21.9, I22.0, I22.1, I22.8, I22.9, I25.2
Cerebral vascular accident	G45.0, G45.1, G45.2, G45.4, G45.8, G45.9, G46.0, G46.1, G46.2, G46.3, G46.4, G46.5, G46.6, G46.7, G46.8, I60.0, I60.1, I60.2, I60.3, I60.4, I60.5, I60.6, I60.7, I60.8, I60.9, I61.0, I61.1, I61.2, I61.3, I61.4, I61.5, I61.6, I61.8, I61.9, I62.0, I62.1, I62.9, I63.0, I63.1, I63.2, I63.3, I63.4, I63.5, I63.6, I63.8, I63.9, I64, I65.0, I65.1, I65.2, I65.3, I65.8, I65.9, I66.0, I66.1, I66.2, I66.3, I66.4, I66.8, I66.9, I67.0, I67.1, I67.2, I67.4, I67.5, I67.6, I67.7, I67.8, I67.9, I68.1, I68.2, I68.8, I69.0, I69.1, I69.2, I69.3, I69.4, I69.8
Peripheral vascular disease	I71.00, I71.01, I71.02, I71.03, I71.1, I71.2, I71.3, I71.4, I71.5, I71.6, I71.8, I71.9, I73.9, I79.0, R02, Z95.8, Z95.9
Based on Sundararajan et al. 2004	
