# Supplementary material for: Predictors of Non-Cardiovascular Readmissions in Multimorbid Adults with Heart Failure in Australian Hospitals: A Retrospective Cohort Study
Source: J Clin Med. 2026 Jul 6;15(13):5275. doi: 10.3390/jcm15135275 (PMC13363480; doi:10.3390/jcm15135275)
Supplement: Supplementary file 1 [file jcm-15-05275-s001.zip › Supplementary Table S1.pdf]

Supplementary Table S1: Principal Diagnoses for First Non-Cardiovascular Readmissions

ICD-10 Group	Number (n)	Percent (%)
Diseases of the respiratory system	550	26.1%
Injury, poisoning and certain other consequences of external causes	256	12.2%
Symptoms, signs and abnormal clinical and laboratory findings, not elsewhere classified	186	8.8%
Certain infectious and parasitic diseases	160	7.6%
Diseases of the digestive system	146	6.9%
Diseases of the blood and blood-forming organs and certain disorders involving the immune mechanism	127	6.0%
Diseases of the genitourinary system	125	5.9%
Endocrine, nutritional and metabolic diseases	108	5.1%
Diseases of the musculoskeletal system and connective tissue	92	4.4%
Mental and behavioural disorders	91	4.3%
Neoplasms	66	3.1%
Diseases of the skin and subcutaneous tissue	61	2.9%
Diseases of the circulatory system	37	1.8%
Diseases of the nervous system	37	1.8%
Miscellaneous	30	1.4%
Diseases of the eye and adnexa	29	1.4%
Diseases of the ear and mastoid process	4	0.2%
